# Impact de la COVID-19 sur la vaccination de routine en milieu hospitalier au Sénégal

**DOI:** 10.11604/pamj.2020.37.364.25805

**Published:** 2020-12-21

**Authors:** Amadou Sow, Modou Gueye, Djibril Boiro, Abou Ba, Idrissa Demba Ba, Papa Moctar Faye, Amadou Lamine Fall, Ousmane Ndiaye

**Affiliations:** 1 Chaire de Pédiatrie, Université Cheikh Anta Diop de Dakar, Fann, Sénégal

**Keywords:** COVID-19, vaccination, impact, enfants, Sénégal, COVID-19, vaccination, impact, children, Senegal

## Abstract

**Introduction:**

la pandémie COVID-19 a poussé le monde à mettre en œuvre des méthodes de prévention drastiques basées sur la limitation des mouvements des populations ayant un impact sur les politiques de santé publique comme la vaccination. L´objectif de ce travail était d´évaluer l´impact de ces mesures de prévention sur la vaccination de routine en milieu hospitalier depuis l´avènement de cette pandémie au Sénégal.

**Méthodes:**

il s´agit d´une étude transversale rétrospective réalisée en août 2020 dans l´unité de vaccination du centre hospitalier Abass NDAO. Nous avons comparé les données de l´unité de vaccination durant la période allant de mars à août des trois dernières années (2018, 2019 et 2020). Le paramètre étudié était le nombre de dose de vaccins administrée pour les différentes périodes selon le programme élargi de vaccination.

**Résultats:**

pour les vaccins administrés à la sixième semaine en avril, le nombre de dose était de 36 en 2018, 29 en 2019 et 15 en 2020 soit une baisse de 50% par rapport au mois de mars. Au mois de juillet le nombre de dose administrée était de 40 en 2018, 35 en 2019 et 15 en 2020 soit une réduction de 42% par rapport à 2019.

**Conclusion:**

les mesures de lutte contre cette pandémie ne devraient pas faire oublier la vaccination de routine surtout dans nos pays à ressources limitées. Il est indispensable de poursuivre la vaccination pour les enfants et d´identifier les enfants qui ont raté des doses vaccinales pour un rattrapage.

## Abstract (en)

**Introduction:**

the COVID-19 pandemic has pushed the world to implement drastic prevention methods based on limiting population movements that have an impact on public health policies, such as vaccination. The purpose of this work was to assess the effect of these preventive measures on routine immunization schedules in hospitals after the outbreak of this pandemic in Senegal.

**Methods:**

we conducted a retrospective cross-sectional study in the Vaccination Unit of the Abass NDAO hospital center in August 2020. We compared data from the Vaccination Unit over the period March-August of the last three years (2018, 2019 and 2020). The parameter studied was the number of vaccine doses administered in the different periods according to the expanded immunization program.

**Results:**

in April, the number of doses of vaccines given at 6 weeks was 36 in 2018, 29 in 2019 and 15 in 2020, reflecting a decrease of 50% compared to March. In July, the number of doses given was 40 in 2018, 35 in 2019 and 15 in 2020, reflecting a reduction of 42% compared to 2019.

**Conclusion:**

measures to combat this pandemic should not affect routine immunization programmes, especially in our resource-constrained country. It is essential to continue vaccination schedule for children and to identify children who have missed vaccine doses in order to implement catch-up vaccination.

## Introduction

La COVID-19 a été identifiée en janvier 2020 comme la cause d'une épidémie de pneumonie touchant en Chine [1]. Elle a été déclarée par l'Organisation Mondiale de la Santé (OMS) comme une pandémie en raison du taux de contagion élevé [2]. A la date du 28 août 2020, la COVID-19 a causé la mort de 833,135 (3.40%) personnes dans le monde et infectées 24 532 835 [3]. Cette pandémie a poussé le monde à mettre en œuvre des méthodes de prévention drastiques basées sur des mesures barrières, l´isolement, la mise en quarantaine et la limitation des mouvements des populations [1]. Ces mesures ont eu un impact considérable très négative sur le plan économique et sur les politiques de santé publique comme la vaccination [4]. Au Sénégal, le programme élargi de vaccination (PEV) (Tableau 1) a été lancé depuis 1974 avec une couverture vaccinale qui a permis d´éradiquer plusieurs maladies infantiles et de réduire la mortalité des enfants de moins de 5 ans [5]. L´objectif de ce travail était d´évaluer l´impact de la COVID-19 et des mesures de prévention sur la vaccination de routine en milieu hospitalier depuis l´avènement de cette pandémie dans le pays le 02 mars 2020.

**Tableau 1:** calendrier du programme élargi de vaccination (PEV) au Sénégal

Ages	Vaccins	Maladies cibles
**A la naissance**	**Hepatite B, BCG, Polio Oral**	Hépatite B, tuberculose, Poliomyélite
**Sixième semaine**	**VPO 1, Pentavalent 1**	Diphtérie
	**Antirotavirus 1**	Tétanos
	**Antipneumococcique 1**	Coqueluche
		Hépatite B
**Dixième semaine**	**VPO 2**	Infections à Hib
	**Pentavalent 2**	
	**Antirotavirus 2**	
	**Antipneumococcique 2**	Poliomyélite
		Infections à Pneumocoque
**Quatorzième semaine**	**VPI**	Infections à Rotavirus
	**Pentavalent 3**	
	**Antipneumococcique 3**	
**Neuvième mois**	**RR1**	Rougeole-Rubéole
	**FJ**	Fièvre jaune
**15 mois**	**RR2**	Rougeole-Rubéole

BCG= bacille Calmet Guerrin, VPO=vaccin poliomyélite oral, VPI=vaccin poliomyélite injectable, FJ=fiàvre jaune, RR=rougeole-rubéole

## Méthodes

Il s´agit d´une étude transversale rétrospective réalisée durant le mois d´août 2020 dans l´unité de vaccination du centre hospitalier Abass NDAO (CHAN) de Dakar qui est un hôpital public de niveau III. L´unité de vaccination est très fréquentée car le CHAN abrite l´un des plus grands pôles mère-enfant du pays avec une maternité qui reçoit en moyenne 5.000 parturientes par année. Tous les nouveau-nés nés dans la structure sont directement orientés vers l´unité de vaccination et sont suivis selon le calendrier vaccinal jusqu´à l´âge de 15 mois. L´unité reçoi également les enfants qui sont nés en dehors de la structure. Nous avons comparé les données de l´unité de vaccination durant la période allant du mois de mars au mois d´août des trois dernières années (2018, 2019 et 2020). Le paramètre étudié était le nombre de dose de vaccins administrée pour les différentes périodes selon le PEV: à la naissance (le BCG, la poliomyélite), à 6 semaines puis à 10 semaines (le pentavalent, le vaccin antipneumococcique, antirotavirus et contre la poliomyélite), à 14 semaines et à 9 mois (rougeole, rubéole et fièvre jaune). Les données ont été recueillies à partir du registre de vaccination et collectées sur une fiche d´enquête préétablie. L´analyse des données a été faite avec la version 9.4 du logiciel SAS.

## Résultats

**Figure 1:**
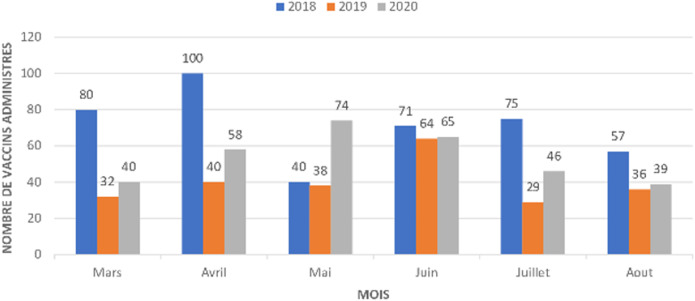
nombre de dose administrée pour les vaccins à la naissance contre (BCG, poliomyélite oral) en fonction du mois de 2018 à 2020

**Figure 2:**
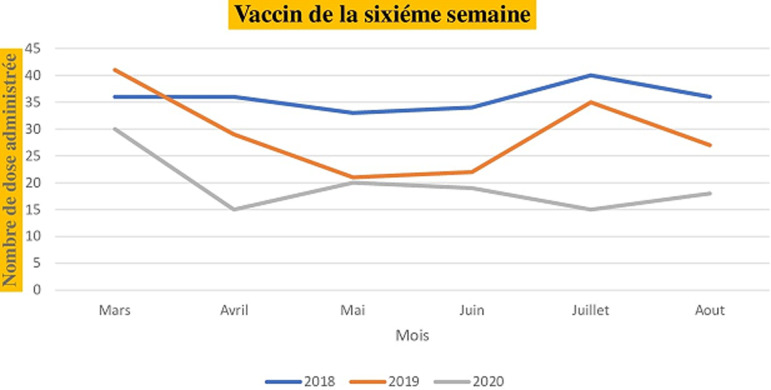
nombre dose administrée pour les vaccins de la sixième semaine en fonction du mois durant les années 2018, 2019 et 2020

**Figure 3:**
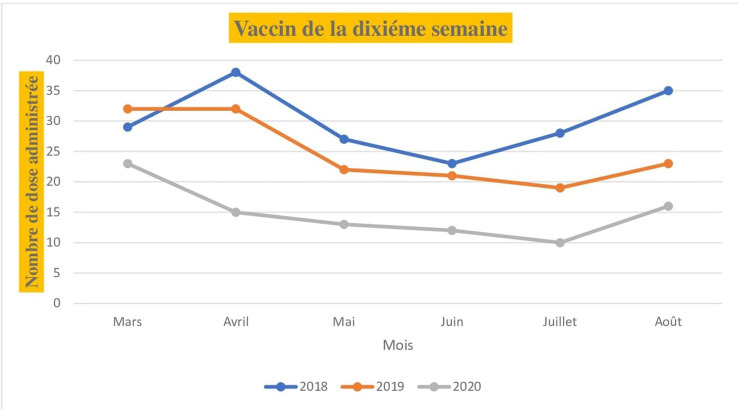
nombre de dose administrée pour les vaccins de la dixième semaine en fonction du mois durant les années 2018, 2019 et 2020

**Figure 4:**
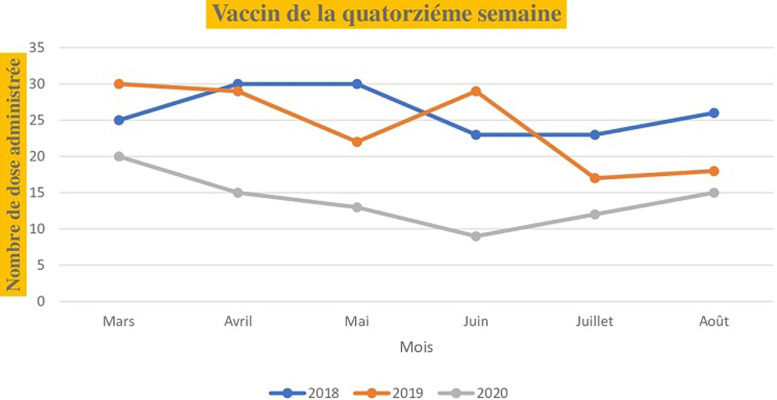
nombre de dose administrée pour les vaccins de la quatorzième semaine en fonction du mois durant les années 2018, 2019 et 2020

**Figure 5:**
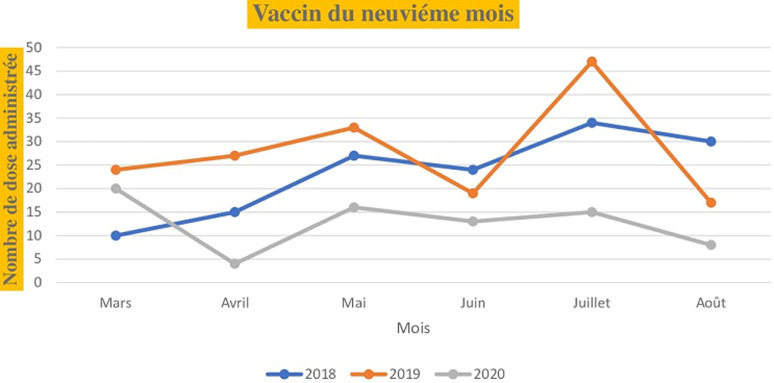
nombre de dose administrée pour les vaccins du neuvième mois en fonction du mois durant les années 2018, 2019 et 2020

Pour les vaccins administrés dès la naissance (la tuberculose, poliomyélite oral), le nombre de dose administrée au niveau de l´unité de vaccination était de 80 pour le mois de mars 2018, 32 pour mars 2019 et 40 pour mars 2020. Les mêmes tendances sont observées en comparant les mois d´avril, de mai, de juin, de juillet et d´août de l´année 2020 aux deux dernières années (2018,2019). Le nombre de dose administrée pour les vaccins à la naissance en fonction du mois et de l´année est illustré dans la Figure 1. Pour les vaccins administrés à la sixième semaine selon le PEV, on note 36 doses administrés au mois de mars 2018, 41 doses en 2019 et 30 doses en 2020. Au mois d´avril, le nombre de dose était de 36 en 2018, 29 en 2019 et 15 en 2020 soit une baisse de 50% par rapport au mois de mars. Au mois de juillet le nombre de dose administrée était de 40 en 2018, 35 en 2019 et 15 en 2020 soit une réduction de 37% par rapport à 2018 et 42% par rapport à 2019. Les nombres de dose administrée pour les vaccins de la sixième, la dixième et les quatorzièmes semaines sont illustrée dans les Figure 2, Figure 3 et Figure 4. Concernant les vaccins contre la fièvre jaune, la rougeole et la rubéole administrés au neuvième mois, le nombre de dose administrée était de 10 en 2018, 24 en 2019 et 20 en 2020. En avril, le nombre de dose administrée en 2020 est passe à 5 contre soit une chute de 20.8% par rapport au mois de mars. Les nombres de dose administrée pour les vaccins du neuvième mois en fonction du mois et de l´année sont illustrés dans la Figure 5.

## Discussion

Au Sénégal, le premier cas de COVID-19 a été déclaré le 2 mars 2020 et quelques semaines après, l´état d´urgence a été déclaré dans le pays limitant ainsi la mobilité de la population. Le nombre de dose administrée pour les vaccins contre la tuberculose et la poliomyélite à la naissance au mois de mars 2020 n´a pas été impacté par la COVID-19. Cela pourrait s´expliquer par le fait que la pandémie n´a pas eu de répercussions sur les accouchements durant cette période et que les mesures d´isolement étaient encore limitées au niveau individuel. Nous avons constaté une nette diminution du nombre de dose administré pour les vaccins de la sixième semaine, de la dixième semaine et de la quatorzième semaine durant la période d´avril à août 2020.

Des résultats similaires ont été observés dans les pays développés [6-8]. Cette période correspond à une augmentation constante du nombre de cas atteint et coïncidant avec la mise en place de mesures de distanciation physique à savoir un couvre-feu, la fermeture des marches et lieu de culte, la réduction du nombre passager dans les transports publics [9]. Cet impact pourrait aussi s´expliquer par le fait que le message sur le fait de rester à la maison a au départ submergé le message que le programme de vaccination devait continuer à fonctionner comme d'habitude et aussi a entrainé des perturbations au niveau des unités de vaccination [1]. La perturbation des services de vaccination pourrait déclencher des flambées secondaires des maladies évitables par la vaccination et aggraver également la longue inégalité dans la couverture vaccinale, surtout dans les zones urbaines [10].

## Conclusion

La COVID-19 a impacté certainement la vaccination de routine des enfants. Les mesures de lutte contre cette pandémie ne devraient pas faire oublier la vaccination de routine surtout dans nos pays à ressources limitées. Il est indispensable de poursuivre ces programmes de vaccination pour les enfants de moins de 5 ans et d´identifier les enfants qui ont raté des doses vaccinales pour un rattrapage. Cela pourrait permettre d´éviter la réapparition de nouvelles épidémies comme la rougeole qui pourrait être associée à une morbi-mortalité élevée.

### Etat des connaissances sur le sujet

- La perturbation des politiques de santé publique comme la vaccination en rapport avec la COVID-19;
- L’importance des mesures de prévention (isolement, distanciation physique) dans la lutte contre la COVID-19.

### Contribution de notre étude à la connaissance

- Les conséquences directes de la pandémie sur l´administration des doses vaccinales chez les enfants vivants dans un pays sous-développé;
- Données sur l´impact de la pandémie dans un pays africain en milieu hospitalier.

## Remerciements

Nous remercions le personnel de l´unité de vaccination en particulier Madame Fall Awa Touré et la direction du centre hospitalier Abass Ndao de Dakar.
